# Physical Activity Guidelines for Astronauts: An Immunological Perspective

**DOI:** 10.3390/biom15101390

**Published:** 2025-09-30

**Authors:** Amirhossein Ahmadi Hekmatikar, Katsuhiko Suzuki

**Affiliations:** 1 Department of Physical Education and Sport Sciences, Faculty of Humanities, Tarbiat Modares University, Tehran 10600, Iran; 2 Faculty of Sport Sciences, Waseda University, Tokorozawa 359-1192, Japan

**Keywords:** physical activity, NASA, immunomodulation, inflammation

## Abstract

Spaceflight imposes unique physiological stressors that profoundly disrupt immune regulation, including impaired lymphocyte activation, latent viral reactivation, and chronic low-grade inflammation. While structured exercise is the cornerstone countermeasure for musculoskeletal and cardiovascular health, current protocols rarely integrate immune endpoints into their design. This review aims to synthesize current evidence on the immunological effects of exercise in spaceflight and propose a novel framework for immune-focused physical activity guidelines tailored to long-duration missions. Evidence indicates that exercise intensity and modality critically determine immune outcomes. Acute strenuous exercise may transiently suppress immunity via cortisol and reactive oxygen species pathways, whereas chronic moderate-to-vigorous training enhances immune surveillance, reduces systemic inflammation, and supports T-cell and NK-cell function. Exerkines such as IL-15, IL-7, and irisin emerge as central mediators of exercise-induced immunomodulation, with potential applications for spaceflight countermeasures. Incorporating immune health into exercise guidelines represents a necessary paradigm shift for astronaut care. A structured framework—emphasizing aerobic, resistance, and HIIT modalities; moderate-to-vigorous intensity; daily training; immune biomarker monitoring; and integration with nutrition and sleep—can enhance resilience against infection, viral reactivation, and cancer risk. Immune-focused countermeasures will be essential to safeguard astronaut health and ensure mission success on future deep-space expeditions.

## 1. Introduction

In recent years, as spaceflight has increasingly expanded in the pursuit of scientific exploration and the advancement of human knowledge, growing concerns have emerged regarding the health of astronauts. Among the most critical of these concerns are alterations in the immune system that occur during space missions and persist upon return to Earth [1]. The immune system is the body’s primary defense against pathogens, toxins, and malignant cells, and any dysregulation can have profound consequences for both astronaut health and the success of space missions [1]. Accumulating evidence indicates that astronauts experience heightened systemic inflammation both during spaceflight and upon reentry (RTE) [2,3,4]. Recent multi-omics approaches integrating transcriptomics, proteomics, and metabolomics from long-duration space missions have revealed coordinated alterations in immune signaling pathways, mitochondrial energy metabolism, and redox balance [5,6,7]. Such datasets provide a system level understanding of how microgravity, radiation, and isolation synergistically modulate immune resilience, offering novel biomarkers for countermeasure development [5,6,7]. Specifically, studies have shown that key inflammatory cytokines involved in early immune responses to infection—including TNF, IL-1, and IL-6—are markedly elevated during spaceflight and RTE [1,2,3,4]. Furthermore, stressors associated with space travel are reported to chronically elevate stress hormone release, which can further compromise immune function [8,9]. Excessive production of reactive oxygen species (ROS) also impairs T-cell function, particularly in CD8+ T cells, facilitating incomplete viral clearance and reactivation of latent viruses [10]. Collectively, these immune disturbances increase astronauts’ susceptibility to infection, viral reactivation, and potentially oncogenic processes [1,2,3,4,8,9]. Recent spaceflight and analog studies have confirmed that microgravity, space radiation, and chronic mission stress synergistically increase ROS production and impair antioxidant defenses, contributing to T-cell dysfunction, NK-cell cytotoxicity loss, and enhanced apoptosis in B cells [11,12,13]. Persistent oxidative stress markers, including glutathione peroxidase 1 downregulation and DNA damage in CD4^+^ T cells, have been documented post-flight, underscoring the need for targeted antioxidant countermeasures in astronaut care.

At present, countermeasures on the International Space Station are largely directed toward preventing musculoskeletal deterioration through structured exercise programs [14,15,16,17,18]. Resistance and aerobic training are known to play pivotal roles in maintaining skeletal muscle and bone integrity, primarily through activation of the AKT/mTOR pathway, which promotes muscle hypertrophy and bone mineral density [19]. Regular resistance exercise, implemented both pre-flight and in-flight, has been shown to substantially mitigate muscle atrophy [14,15,16,17,18]. Nevertheless, while exercise is recognized for its musculoskeletal benefits, research specifically addressing its role in modulating immune alterations during spaceflight remains scarce.

Although exercise is already integrated as a core countermeasure in current space missions, insufficient attention has been devoted to its potential immunomodulatory effects. This knowledge gap has important implications for astronaut health, particularly in the context of long-duration missions beyond low Earth orbit. Accordingly, the aim of this review is to synthesize current evidence on immune dysregulation in astronauts, critically evaluate the role of exercise interventions in modulating these changes, and ultimately propose an exercise-based framework to mitigate immune dysfunction during space travel.

## 2. Immune Dysregulation in Spaceflight

### 2.1. Viral Reactivation Under Microgravity

Microgravity, a defining condition of spaceflight, profoundly alters immune system function. Evidence from long-duration missions indicates that immune cell proliferation, differentiation, and activation are consistently impaired. These alterations not only weaken host defenses against pathogens but also disrupt immune homeostasis [1]. Extensive research has examined the impact of microgravity on various immune cell subsets, including macrophages, dendritic cells, neutrophils, natural killer (NK) cells, and T and B lymphocytes. Findings demonstrate that microgravity influences immune cells at both molecular and functional levels, leading to broad dysregulation of immune responses [1]. One clinically significant outcome of this dysregulation is the reactivation of latent herpesviruses. Studies reveal that Epstein–Barr virus (EBV), Varicella–Zoster virus (VZV), and Cytomegalovirus (CMV) reactivate in over half of astronauts, with higher prevalence during extended missions [1]. Although viral reactivation is usually asymptomatic, it may persist after return to Earth and pose health risks for immunocompromised individuals, particularly those on Earth whom astronauts may come into contact with after returning from space missions. The underlying mechanism involves suppression of cellular immune responses, further exacerbated by stress-induced elevations in cortisol, epinephrine, and norepinephrine, as well as impaired T-cell and NK-cell activity. Consequently, microgravity is now recognized as a key contributor to both immune dysregulation and viral reactivation [1]. The clinical relevance of these findings extends beyond space medicine. Technologies developed for in-flight viral monitoring have been translated into terrestrial applications, particularly for the early detection of immune dysfunction and prevention of virus-related diseases. For example, studies report that during short shuttle missions (10–16 days), approximately 53% of astronauts exhibited herpesvirus reactivation—most commonly EBV, VZV, HSV-1, or CMV—in saliva or urine. On long-duration missions aboard the International Space Station (>180 days), this prevalence rises to approximately 61% [20]. While reactivation is typically asymptomatic, infectious virus has occasionally been isolated and linked to clinical manifestations such as atopic dermatitis and skin lesions [20]. Taken together, these observations highlight the dual influence of microgravity and neuroendocrine stress on immune surveillance, creating conditions conducive to the reactivation of latent viruses. Understanding these mechanisms not only informs countermeasure development for spaceflight but also advances clinical approaches to viral monitoring and immune health on Earth.

### 2.2. Mechanistic Insights into Microgravity-Induced Immune Dysregulation

The mechanisms underlying immune alterations during spaceflight are multifactorial. Microgravity disrupts cellular cytoskeletal architecture, impairing immune cell polarization, migration, and interaction with other cells [21]. Altered fluid shear forces and redistribution of body fluids to the thoracic region may influence lymphocyte trafficking and antigen presentation [22]. Additionally, microgravity modifies intracellular signaling pathways, including NF-κB, JAK–STAT, and MAPK, leading to altered cytokine transcription profiles [12]. Chronic activation of the hypothalamic–pituitary–adrenal axis under spaceflight stress increases glucocorticoid release, suppressing immune activation and favoring anti-inflammatory responses [23]. Moreover, mitochondrial dysfunction and excess production of reactive oxygen species (ROS) under microgravity and space radiation synergistically contribute to systemic inflammation and impaired immunity [24]. These interrelated mechanisms collectively explain why spaceflight can simultaneously elevate systemic inflammatory markers while suppressing effective innate and adaptive immune responses.

### 2.3. Effects on Antigen-Presenting Cells (Dendritic Cells)

As professional antigen-presenting cells, dendritic cells (DCs) serve as a critical bridge between innate and adaptive immunity [25]. Studies conducted under simulated microgravity have reported inconsistent findings regarding DC differentiation and abundance; while some investigations observed a decrease, others reported an increase [25,26]. The duration of microgravity exposure and the simulation method appear to be key determinants of these outcomes [26]. An increase in DC numbers under weightlessness has been associated with impaired antigen-specific T-cell activation, mediated through reduced interleukin-2 (IL-2) secretion. This decline in IL-2 not only weakens T-cell responses but also limits the generation of regulatory T cells, thereby increasing susceptibility to autoimmunity and inflammation (3). Conversely, evidence suggests that prolonged microgravity exposure suppresses DC maturation and diminishes their capacity to effectively stimulate T lymphocytes [25,26,27].

### 2.4. Neutrophils and Innate Immunity

Neutrophils, derived from the bone marrow, represent the first line of defense against invading pathogens. They rapidly migrate to sites of infection, where they exert antimicrobial effects through chemotaxis, phagocytosis, and bactericidal activity [28]. As key mediators of innate immunity, neutrophils are essential for infection control and therefore play a critical role in maintaining astronaut health during space missions [28]. Findings from blood samples collected from 25 astronauts across four missions revealed an increase in both the number and proportion of circulating neutrophils [29]. Similarly, one study reported elevated neutrophil counts and functional alterations following spaceflight [29]. Moreover, the neutrophil-to-lymphocyte ratio (NLR) has emerged as a useful biomarker for monitoring astronaut health, with significant implications for predicting inflammation and cancer risk [30]. Current evidence indicates that microgravity consistently elevates neutrophil counts, likely due to enhanced mobilization from the bone marrow and increased secretion of cytokines such as interleukin-8 (IL-8). However, functional capacity appears compromised under microgravity conditions: processes such as phagocytosis and oxidative burst may be impaired with prolonged exposure, ultimately diminishing neutrophil-mediated host defense [28,29,30]. While most studies confirm an increase in circulating neutrophil counts during and after spaceflight, the magnitude of this rise often remains within the upper limit of the clinically normal range for healthy adults (typically 1.5–8.0 × 10^9^ cells/L). For example, [22,31] reported post-flight neutrophil counts elevated compared to pre-flight baselines but not reaching thresholds indicative of neutrophilia (>8.0 × 10^9^ cells/L) in the majority of astronauts. However, subtle changes in functional capacity—such as diminished oxidative burst and impaired phagocytosis—occurred despite counts being within normal ranges, suggesting that numerical increases may not equate to enhanced immune competence [31]. In some individuals, transient counts at or slightly above the normal range were noted immediately post-landing, potentially reflecting acute stress or inflammatory responses, but these typically normalized within days.

### 2.5. Natural Killer Cells and Tumor Surveillance

Natural killer (NK) cells, derived from bone marrow lymphoid progenitors, rely on both the bone marrow and thymic microenvironment for their differentiation and expansion. Unlike T and B lymphocytes, NK cells can directly recognize and eliminate tumor or virus-infected cells without prior sensitization, making them critical effectors of innate immunity and key mediators of tumor and viral surveillance [32,33]. Findings regarding NK cell dynamics in microgravity remain inconsistent. However, the majority of studies indicate that microgravity decreases NK cell survival while promoting apoptosis [9,32,33]. Consequently, both the total number of NK cells and their cytotoxic activity are diminished. Such impairments may substantially compromise immune surveillance, limiting the body’s ability to control latent viral reactivation or suppress malignant transformation [9,32,33].

### 2.6. B Cells and Humoral Immunity

B lymphocytes, differentiated from pluripotent stem cells in the bone marrow, account for approximately 10–15% of peripheral blood lymphocytes and represent the only immune cells capable of producing antibodies. Through surface antigen-recognition receptors, B cells recognize epitopes on soluble protein antigens, triggering their activation, proliferation, and differentiation into plasma cells that synthesize and secrete diverse immunoglobulins [34]. Evidence suggests that microgravity adversely affects humoral immunity. Post-spaceflight data from amphibians revealed alterations in immune cell counts, while studies in mice exposed to one month of spaceflight aboard biological satellites demonstrated a 41% reduction in splenic B cell numbers [35]. Animal models consistently show that microgravity decreases B cell abundance, likely through mechanisms involving oxidative stress and the generation of reactive oxygen species, which activate apoptosis-inducing pathways [36,37]. However, findings from long-duration missions aboard the International Space Station (ISS) suggest that B cell homeostasis can be maintained under certain conditions, underscoring the complexity of microgravity effects and their dependence on environmental factors and duration of exposure [36,37]. Research on humoral immunity in space remains limited, partly due to the technical challenges of collecting and analyzing antibodies and other soluble mediators in microgravity.

### 2.7. T Cells and Adaptive Immunity

T lymphocytes, derived from thymic progenitors, mature in the thymus and subsequently circulate through lymphoid and peripheral tissues to orchestrate cellular immunity and maintain immune homeostasis. They represent a central component of adaptive immunity. Under microgravity, however, T cell proliferation and activation are markedly impaired [38]. Expression of activation markers such as CD25 and CD69 is reduced, alongside diminished secretion of cytokines including IL-2 and IFN-γ [3,39].

Molecular analyses reveal that microgravity disrupts key signaling cascades, notably the NF-κB and JAK–STAT pathways, thereby attenuating T cell responsiveness. Both CD4^+^ and CD8^+^ T cells exhibit impaired activation under strong stimuli, with reduced CD25, CD69, and JAK–STAT activity, while regulatory T cell (Treg) immunosuppressive responses are enhanced through STAT5-associated signaling [40]. In addition, alterations in cytoskeletal organization and defective trafficking of protein kinase C (PKC) further suppress T cell function [36,41,42]. These changes collectively undermine adaptive immunity during spaceflight and carry important implications for host defense in extraterrestrial environments.

### 2.8. Metabolic and Structural Changes in Immune Cells

**Figure 1:**
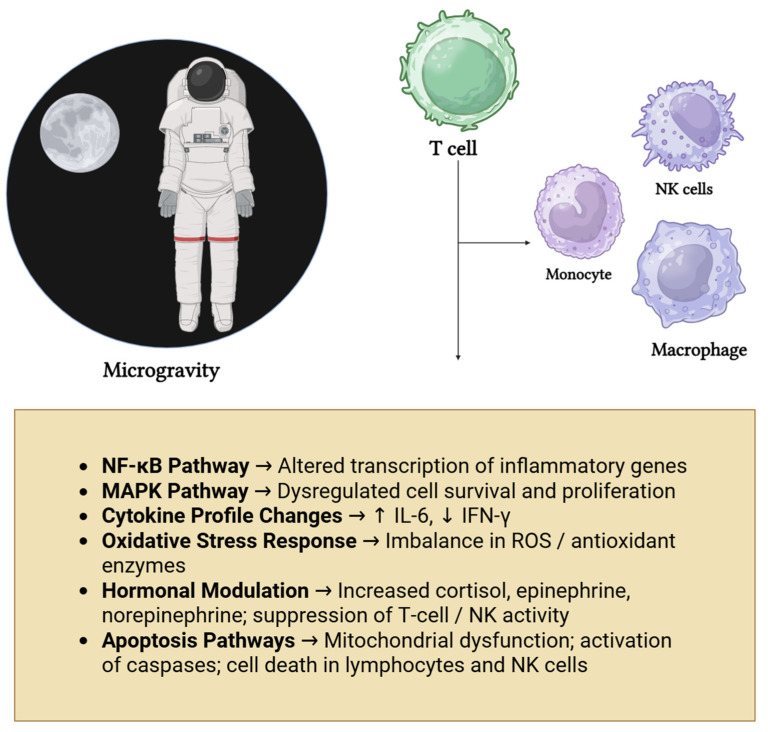
Conceptual overview of key immune signaling pathways affected by microgravity. Exposure to microgravity leads to biomechanical and physiological alterations, which influence multiple immune cell types and disrupt major signaling pathways (NF-κB, MAPK, oxidative stress responses). These molecular changes contribute to impaired immune function and increased susceptibility to disease. Figure 1 created by the authors.

Under normal physiological conditions, macrophages are versatile effector cells of the innate immune system responsible for detecting, engulfing, and eliminating pathogens as well as clearing dead or damaged cells. They arise from circulating monocytes and reside in virtually all tissues, where they act as sentinels and first responders to infectious or inflammatory insults [43]. Beyond their well-known role in phagocytosis, macrophages produce a wide array of cytokines and chemokines to orchestrate immune responses, recruit additional immune cells, and regulate tissue repair and remodeling. They also serve as professional antigen-presenting cells by displaying pathogen-derived peptides via major histocompatibility complex class II (MHC-II) molecules to activate T lymphocytes, thus bridging innate and adaptive immunity [44]. Depending on environmental cues, macrophages can adopt distinct functional phenotypes, ranging from the pro-inflammatory M1 type to the anti-inflammatory and tissue-repairing M2 type, maintaining immune homeostasis and contributing to wound healing. A substantial portion of research on immune cell metabolism under microgravity has focused on macrophages. Findings indicate that lipid and nucleotide metabolic pathways are particularly affected [45,46,47,48]. For example, elevated levels of glycerol-3-phosphate and reduced levels of N-acetyltryptophan have been shown to compromise macrophage capacity to counteract pathogens. Such metabolic alterations not only impair cellular functions but also disrupt overall immune homeostasis. These observations are aligned with recent NASA-led multi-omics profiling studies [5,49], which report lipidome remodeling, disrupted amino acid metabolism, and altered cytokine–metabolite networks under spaceflight and simulated microgravity conditions. Beyond metabolic changes, microgravity induces profound structural modifications in immune cells [48]. Monocytes are circulating leukocytes of the innate immune system derived from bone marrow progenitor cells. They serve as versatile precursors that can differentiate into macrophages or dendritic cells upon migrating into tissues. Under normal physiological conditions, monocytes patrol the bloodstream, rapidly infiltrating sites of infection or injury in response to chemotactic signals [50]. They contribute to host defense through phagocytosis of pathogens, secretion of pro-inflammatory cytokines (e.g., TNF-α, IL-1β, IL-6), and regulation of subsequent adaptive immune responses. Monocytes also participate in tissue remodeling and wound healing, producing growth factors and anti-inflammatory mediators when inflammation subsides. Functionally, they can be categorized into classical (CD14++CD16−), intermediate (CD14++CD16+), and non-classical (CD14+CD16++) subsets, each with specialized roles in immunity, inflammation, and vascular health [51]. In monocytes, reduced expression of surface molecules such as CD62L and HLA-DR interferes with adhesion and migration. Similarly, alterations in cytoskeletal components, including F-actin and microtubules, impair both motility and phagocytosis. In macrophages, decreased expression of ICAM-1 and MHC-II limits antigen presentation and migratory capacity [51]. These structural alterations are frequently associated with inhibition of key signaling pathways, notably protein kinase C (PKC) and p38 MAPK [1,46,48]. Taken together, current evidence demonstrates that microgravity exerts widespread effects on the immune system, disrupting proliferation, differentiation, activation, metabolism, and cellular architecture across multiple immune cell types. Such alterations may heighten susceptibility to infection and immune dysregulation during long-duration spaceflight. A deeper understanding of these mechanisms, along with the development of targeted countermeasures, will be essential for safeguarding astronaut health on future missions (Figure 1).

### 2.9. Space Radiation and Cancer Risk

During spaceflight, astronauts are chronically exposed to ionizing radiation, which poses significant health risks ranging from acute radiation sickness to long-term carcinogenesis [52,53,54]. Of particular concern is galactic cosmic radiation (GCR), composed largely of high-energy and heavy (HZE) particles with strong penetrative capacity. These particles induce complex and clustered DNA lesions that often exceed normal cellular repair mechanisms, thereby increasing the likelihood of cellular transformation and tumorigenesis [52,53,54]. The risk of cancer from space radiation depends not only on dose but also on radiation type and quality. Traditional risk assessment models, such as the linear no-threshold (LNT) model, assume that any dose confers a proportional cancer risk. However, high-linear energy transfer (LET) radiation encountered in deep space produces nonlinear, non-targeted effects that may amplify risk well beyond LNT-based predictions [52,53,54]. NASA risk models for missions up to one year in low-Earth orbit (LEO) suggest that the associated increase in cancer risk remains within acceptable limits. Yet for longer missions (>18–24 months), the 95% confidence interval for radiation exposure–induced death (REID) surpasses acceptable thresholds, exceeding 18 months for women and 24 months for men [45]. NASA currently applies a conservative 3% upper bound on lifetime attributable cancer mortality risk; however, projections for long-duration missions—such as exploratory missions to Mars—approach or exceed this threshold, particularly when accounting for the effects of HZE particles that existing models cannot fully capture [52,53,54]. Evidence from historical astronaut cohorts, though limited, suggests an elevated incidence of cancer with increasing cumulative radiation dose. Furthermore, advanced NASA modeling underscores the significant risks associated with extended missions beyond Earth’s magnetosphere, not only for cancer but also for cardiovascular disease and other late-onset conditions [52,53,54]. Overall, space radiation remains one of the most critical hazards for astronaut health. Its potential to induce DNA damage, cellular transformation, and late-stage malignancies necessitates careful consideration in mission planning. Effective protective and preventive strategies must therefore be prioritized, particularly in the context of future long-duration deep-space missions, including expeditions to Mars (Table 1).

### 2.10. Terrestrial Analogs for Microgravity Simulation

Terrestrial analogs provide indispensable platforms for dissecting microgravity-induced physiological mechanisms under controlled conditions. The −6° head-down tilt bed rest (HDTBR) model, lasting from 30 to 90 days, reproduces cephalad fluid shifts and musculoskeletal unloading observed in space. Recent studies have demonstrated that prolonged HDTBR impairs neutrophil oxidative burst, reduces T-cell receptor activation, and elevates reactive oxygen species (ROS) production [55,56]. Two-dimensional clinostats employ continuous rotation to average the gravity vector, allowing long-term culture of immune cells under simulated weightlessness. This approach has revealed altered dendritic cell maturation, natural killer (NK) cell cytotoxicity, and cytoskeletal dynamics [57]. Random Positioning Machines (RPMs) utilize rapid, computer-controlled multi-axis rotation, closely mimicking microgravity for smaller samples. Transcriptomic and metabolomic analyses have reported shifts in mitochondrial bioenergetics and oxidative stress pathways in lymphocytes following RPM exposure [55,56]. Collectively, these models bridge the gap between in-flight and laboratory research, enabling precise mechanistic studies and the testing of targeted countermeasures, including immune-focused exercise interventions. 

### 2.11. Countermeasures (Exercise) in Spaceflight

Recent studies have raised concerns that spaceflight significantly compromises physical fitness, with notable effects including skeletal muscle atrophy, bone demineralization, and cardiovascular dysfunction [58,59]. To mitigate these risks, space agencies have prioritized structured physical activity as a central countermeasure, both pre-flight and during missions. Understanding how exercise, alone or in combination with other therapeutic modalities, can attenuate these physiological changes is critical for the success of future long-duration human missions to Mars [58,59].

Protocols currently implemented on the International Space Station (ISS) include resistive and aerobic exercise regimens using devices such as the Advanced Resistive Exercise Device (ARED), treadmill, and cycle ergometer [60,61]. These interventions have been shown to mitigate muscle atrophy and support cardiovascular health, representing a cornerstone of astronaut healthcare strategies [60,61].

### 2.12. Psychosocial Stressors, Isolation, and Circadian Disruption in Spaceflight Immunity

Long-duration spaceflight imposes unique psychosocial and environmental stressors that profoundly influence immune regulation. Chronic psychosocial stress, exacerbated by mission demands, confinement, and high workload, activates the HPA axis and sympathetic nervous system, increasing circulating cortisol and catecholamines, which in turn suppress T-cell and NK-cell activity [11]. Social isolation reduces interpersonal interaction and sensory stimulation, factors linked in terrestrial analog studies (e.g., Antarctic winter-over, HERA) to altered cytokine production, diminished NK-cell cytotoxicity, and elevated risk of latent herpesvirus reactivation [62]. Moreover, the absence of natural light–dark cycles induces circadian misalignment, disrupting the central and peripheral clocks that regulate immune and endocrine rhythms. Astronauts frequently experience sleep fragmentation and reduced melatonin production, which are associated with impaired adaptive immunity and pro-inflammatory bias [11]. Together, these factors create a synergistic burden on immune surveillance, warranting targeted behavioral, lighting, and exercise strategies to mitigate their impact in future mission designs.

### 2.13. Advanced Resistive Exercise Device (ARED)

ARED replicates traditional resistance training (e.g., free weights) by providing constant force throughout the range of motion [63]. It enables astronauts to perform both upper- and lower-body exercises—including squats, deadlifts, heel raises, bicep curls, and bench presses—using vacuum cylinders that deliver concentric loads up to 270 kg, with eccentric loads reaching 90% of the concentric force [63]. In addition, ARED offers real-time feedback to astronauts while transmitting performance data to NASA exercise physiologists [63]. Flight surgeons and trainers anticipate that the higher loading capacity of ARED enhances training efficiency and effectiveness, thereby preventing the musculoskeletal decline often observed during extended missions [63]. Empirical evidence supports this role. For example, one study reported that 16 weeks of ARED training significantly increased astronaut strength and muscle mass [14]. Another investigation highlighted ARED’s unique ability to prevent muscle atrophy during spaceflight [64]. Resistance training, including ARED exercise, is among the most effective strategies for promoting muscle hypertrophy, defined as the growth and enlargement of muscle fibers [65].

The molecular mechanisms underlying ARED-induced hypertrophy are primarily linked to the PI3K–AKT–mTOR signaling pathway. The binding of insulin-like growth factor 1 (IGF-1) to its receptor induces receptor autophosphorylation, followed by phosphorylation of insulin receptor substrate-1 (IRS-1), leading to PI3K activation. This pathway promotes downstream activation of AKT and subsequent increases in muscle mass [66,67,68,69,70]. Early evidence demonstrated that PI3K activation alone was sufficient to drive IGF-1–mediated muscle hypertrophy [66,67,68,69,70]. Conversely, the mTOR pathway requires amino acid availability for activation through phosphorylation of S6K1 and 4E-BP1. Unlike insulin, amino acids cannot activate PI3K or PKB directly [19,71]. Recent studies suggest that amino acids may regulate mTOR indirectly via TSC, GBL–raptor, or Rheb signaling, while also promoting mTOR–raptor association, thereby enhancing phosphorylation of 4E-BP1 and S6K1 [66,67,68,69,70].

Collectively, current evidence indicates that ARED, as a form of resistance training, effectively counters spaceflight-induced musculoskeletal decline by activating anabolic signaling pathways. As such, ARED represents a critical countermeasure for mitigating muscle atrophy and bone density loss during long-duration missions.

### 2.14. Treadmills and Cycle Ergometers

Another key countermeasure employed by space agencies is the use of treadmills and cycle ergometers [72,73]. Treadmill exercise has been a standard intervention since the Mir program and has continued on the Space Shuttle and the International Space Station (ISS) as a countermeasure against musculoskeletal and cardiovascular deconditioning during spaceflight [72,73]. Similarly to the original treadmill designed by Dr. William Thornton and tested aboard the Space Shuttle [74], all spaceborne treadmills require crewmembers to be tethered via Subject Load Devices (SLDs)—elastic bungees or other loading systems—that provide a “gravity replacement” force to keep the astronaut in contact with the belt [74]. Treadmill and cycle ergometer exercise combine aerobic and resistive elements, which contribute to both improved cardiovascular performance and muscle hypertrophy. For example, Lee et al. demonstrated that integrating aerobic exercise with resistance training significantly enhances muscle hypertrophy [75]. Additional studies have confirmed that moderate-intensity aerobic exercise improves cardiovascular function, highlighting its role as a protective strategy during long-duration missions [76,77,78]. Despite these benefits, a critical gap remains in the literature: most studies have prioritized musculoskeletal and cardiovascular outcomes, with relatively little attention given to the immunological adaptations to treadmill and cycle ergometer exercise in microgravity. Addressing this gap will be essential for a comprehensive understanding of exercise as a multi-system countermeasure. Collectively, NASA/ESA exercise countermeasures have been highly successful in mitigating musculoskeletal and cardiovascular decline during spaceflight. However, a major scientific gap remains: the immune system is rarely considered as a primary endpoint. Future countermeasure design must therefore expand beyond bone and muscle to encompass immune resilience, particularly in the context of infection risk, latent viral reactivation, and cancer susceptibility during long-duration missions (Table 2).

For example, recent ISS and ground-based analog studies indicate that vitamin D supplementation (targeting serum 25(OH)D > 75 nmol/L) can modulate T-cell responses and reduce systemic inflammation during microgravity exposure [1,79]. Probiotic supplementation has been shown to support gut–immune homeostasis, enhance NK-cell activity, and reduce frequency of viral reactivation episodes in crewmembers [80]. Additionally, studies of pharmacological immunomodulators such as low-dose IL-7 or A2A receptor agonists have highlighted potential synergistic effects with aerobic or resistance training in restoring lymphocyte counts and cytokine balance during analog models [81].

### 2.15. Exercise & Immune Function: Evidence from Earth

Exercise has long been recognized as a powerful modulator of immune function, exerting effects that can vary depending on intensity, duration, and frequency. The distinction between acute and chronic exercise is essential for understanding the dual nature of exercise-induced immune modulation. Acute exercise refers to a single bout of physical activity that elicits transient changes in immune cell trafficking, cytokine secretion, and stress hormone release. In contrast, chronic exercise—defined as repeated bouts over weeks to months—induces long-term adaptations, enhancing immune resilience and reducing inflammation [82,83]. This dichotomy provides a conceptual framework for investigating exercise immunology. Evidence from terrestrial studies suggests that moderate-intensity exercise—approximately 45–60 min per session, performed 3–5 days per week at 50–70% of VO_2_max—optimally enhances immune surveillance, regulates inflammation, and lowers the risk of infection. In contrast, prolonged high-intensity training (>90 min at >75% VO_2_max) without sufficient recovery can induce an “open window” of transient immune suppression, lasting from 3 to 72 h, characterized by reduced NK cell activity, lymphocytopenia, and increased susceptibility to upper respiratory tract infections (URTI) [83]. This phenomenon is often observed in overreaching or overtraining syndromes, where cumulative physiological and psychological stressors exceed the body’s adaptive capacity. Periodizing training loads and ensuring adequate recovery—through rest days, sleep optimization, and nutritional support—are therefore critical to maximizing the immunological benefits of exercise while avoiding detrimental effects [84].

### 2.16. Acute Exercise: Transient Mobilization and Stress-Related Immunity

During acute exercise, stress hormones such as epinephrine, norepinephrine, and cortisol rise rapidly. These catecholamines bind to β2-adrenergic receptors on leukocytes, activating cyclic AMP (cAMP)–protein kinase A (PKA) signaling. This triggers downstream activation of Rho GTPases and cytoskeletal remodeling, facilitating leukocyte demargination from vessel walls into circulation [82,85]. As a result, the number of circulating NK cells, CD8+ T lymphocytes, and neutrophils increases two- to fourfold within minutes of starting exercise. NK cell mobilization is particularly sensitive to epinephrine due to high β2-receptor expression. Functionally, this acute surge enhances immune surveillance, improving detection of virally infected and malignant cells [85]. However, after exercise cessation, cortisol-mediated signaling through the glucocorticoid receptor (GR) activates transcriptional repressors of IL-2 and IFN-γ, leading to transient lymphopenia [86]. This post-exercise “open window” has been associated with increased susceptibility to upper respiratory tract infections (URTIs), although the clinical significance remains debated. Acute exercise induces the release of cytokines, primarily IL-6, IL-1β, and TNF-α. Among these, IL-6 is the most prominent and is classified as both a pro-inflammatory cytokine and an exercise-induced myokine [82]. During muscle contraction, Ca^2+^ influx activates CaMK and p38 MAPK, which in turn phosphorylate transcription factors such as NF-κB and AP-1, promoting IL-6 gene expression. Thus, acute exercise creates a transient pro- to anti-inflammatory shift, highlighting the complexity of IL-6 as both a marker of stress and a mediator of recovery [82]. Acute strenuous exercise increases the production of reactive oxygen species (ROS) through mitochondrial electron leakage, xanthine oxidase activity, and NADPH oxidase activation. Excessive ROS activate NF-κB and p38 MAPK, promoting pro-inflammatory cytokine expression [87]. At the same time, oxidative stress can impair T-cell receptor (TCR) signaling by oxidizing Lck kinase and CD3ζ chains, thereby suppressing adaptive immunity [88]. These findings explain why acute high-intensity or prolonged endurance exercise may transiently compromise immune competence, particularly when combined with inadequate recovery [88].

### 2.17. Chronic Exercise: Adaptation, Resilience, and Immune Homeostasis

Unlike acute bouts, chronic exercise training is consistently associated with reductions in systemic low-grade inflammation. Repeated activation of anti-inflammatory pathways during each exercise session contributes to cumulative downregulation of inflammatory signaling. Specifically, chronic aerobic and resistance training attenuate Toll-like receptor 4 (TLR4) expression on monocytes, reducing downstream NF-κB activation and TNF-α production [89]. Moreover, sustained improvements in antioxidant defenses (e.g., upregulation of superoxide dismutase and glutathione peroxidase) reduce ROS-induced immune dysregulation [89]. Aging is associated with immunosenescence, characterized by reduced naïve T-cell output, expansion of memory CD8+ T cells, and impaired vaccine responses. Chronic exercise mitigates these changes by enhancing thymic output and preserving T-cell receptor diversity [88]. Mechanistically, exercise reduces levels of senescence-associated secretory phenotype (SASP) cytokines (IL-6, TNF-α), which otherwise promote T-cell exhaustion. Additionally, regular training modulates AMPK–mTOR signaling, supporting mitochondrial biogenesis in T-cells and improving their metabolic fitness [88]. Unlike acute transient increases, long-term training enhances the baseline functional capacity of NK cells. Exercise-induced IL-15 secretion from skeletal muscle plays a critical role by promoting NK-cell proliferation and survival through JAK–STAT5 signaling [90]. These adaptations translate into clinically relevant outcomes, such as improved viral clearance and enhanced anti-tumor immunity in both animal models and humans [91]. While acute bouts of exercise increase cortisol and catecholamines, chronic training results in adaptations of the hypothalamic–pituitary–adrenal (HPA) axis. Resting cortisol levels decrease, and stress-induced spikes are attenuated, reducing glucocorticoid-mediated immune suppression [92].

This endocrine rebalancing supports long-term immune resilience by minimizing chronic stress-related immune dysregulation. The immune system’s response to exercise is profoundly shaped by whether the stimulus is acute or chronic. Acute bouts mobilize immune cells and transiently induce cytokine and stress responses, which may enhance immediate surveillance but can suppress function during recovery. By contrast, chronic training exerts cumulative anti-inflammatory and immune-restorative effects, mediated through adaptations in redox balance, signaling pathways (NF-κB, AMPK–mTOR, JAK–STAT), and neuroendocrine regulation. These findings underscore the importance of distinguishing between acute and chronic effects when designing exercise interventions for immune health, particularly in populations vulnerable to infection, immunosenescence, or chronic inflammation (Table 3).

### 2.18. Exerkines as Immune Mediators

The term exerkines refers to bioactive molecules secreted into circulation in response to physical exercise. These factors include myokines (muscle-derived), adipokines (adipose tissue–derived), cardiokines (cardiac-derived), hepatokines (liver-derived), osteokines (bone-derived), and neurokines (neuron-derived) [93,94]. Exerkines function as systemic messengers, orchestrating crosstalk between contracting skeletal muscle and distant organs, including the immune system [95]. The discovery of exerkines has transformed our understanding of exercise immunology. Far beyond simple hemodynamic or mechanical effects, exercise induces a paracrine and endocrine signaling network that modulates leukocyte function, cytokine balance, and inflammatory resolution. These signaling networks are mediated through canonical pathways such as JAK–STAT, PI3K–AKT–mTOR, AMPK–PGC1α, and NF-κB [95].

1. Myokines: The Muscle–Immune Interface

IL-6 is the prototypical myokine, released in large quantities during muscle contraction. Its secretion is triggered by intracellular Ca^2+^ flux, AMPK activation, and p38 MAPK signaling [82,93]. Unlike pro-inflammatory IL-6 released from immune cells, exercise-induced IL-6 acts primarily in an anti-inflammatory manner (1) Activates JAK–STAT3 signaling in hepatocytes → induces IL-1 receptor antagonist (IL-1ra) and IL-10, both of which suppress TNF-α and (2) Promotes lipolysis and glucose uptake, indirectly reducing metabolic inflammation [82,93]. IL-6 also regulates NK-cell trafficking by upregulating adhesion molecules (ICAM-1, VCAM-1) on endothelial cells, facilitating NK recruitment to peripheral tissues [82,93]. Both IL-7 and IL-15 are essential for T-cell homeostasis and NK-cell development. (1) IL-7: Promotes thymopoiesis and naïve T-cell survival via JAK1/3–STAT5 signaling [96]. Chronic training increases basal IL-7 secretion, potentially counteracting immunosenescence [96]. Also, IL-15 Strongly upregulated in skeletal muscle after resistance exercise. IL-15 binds to the IL-15Rα/IL-2Rβ/γc complex, activating JAK1/3–STAT3/5 pathways. This enhances NK-cell proliferation and cytotoxicity and supports CD8+ memory T-cell survival [90].

Irisin, a cleavage product of FNDC5, is induced by PGC1α activation during exercise. Irisin exerts immunomodulatory effects by: (1) Reducing pro-inflammatory cytokine production in macrophages via inhibition of NF-κB and MAPK signaling, (2) Enhancing regulatory T-cell (Treg) differentiation, contributing to immune tolerance AMD (3) Promoting “browning” of adipose tissue, thereby reducing adipose-related inflammation [95,97]. In contrast to anabolic myokines, myostatin is a negative regulator of muscle mass. Exercise downregulates myostatin, thereby releasing inhibition on muscle hypertrophy. Immunologically, myostatin upregulates TGF-β–SMAD2/3 signaling, which promotes fibrosis and immune suppression. Thus, exercise-induced suppression of myostatin indirectly supports anti-inflammatory balance [95].

2. Adipokines: Exercise and Immunometabolism

Leptin, secreted from adipocytes, exerts pleiotropic effects on immune cells. It promotes Th1 responses and pro-inflammatory cytokine secretion (IL-2, IFN-γ, TNF-α) via JAK2–STAT3 and PI3K–AKT pathways [95,98]. Chronic exercise reduces leptin levels, dampening its pro-inflammatory impact and lowering risk of autoimmunity. Adiponectin is generally anti-inflammatory, activating AMPK–PPARα signaling to inhibit NF-κB activation in macrophages and endothelial cells. Exercise consistently elevates adiponectin, contributing to reduced chronic inflammation [99]. Beyond leptin and adiponectin, adipose tissue secretes a wide spectrum of cytokines—collectively known as adipokines—that exert predominantly pro-inflammatory actions and can modulate immune function. These include tumor necrosis factor-alpha (TNF-α), interleukin-6 (IL-6), monocyte chemoattractant protein-1 (MCP-1/CCL2), resistin, visfatin, and omentin. TNF-α and IL-6 contribute to systemic low-grade inflammation, impair insulin signaling, and promote T-cell activation, which may exacerbate immune dysregulation under microgravity [100]. MCP-1 recruits monocytes and macrophages into adipose tissue, amplifying local and systemic inflammation. Resistin and visfatin have been implicated in metabolic stress–induced immune activation, whereas omentin possesses anti-inflammatory properties, highlighting the heterogeneity of adipokine effects. The balance between these pro- and anti-inflammatory signals from adipose tissue may influence astronauts’ immune resilience during prolonged space missions [101].

3. Cardiokines and Hepatokines

Cardiac contraction releases atrial natriuretic peptide (ANP) and B-type natriuretic peptide (BNP), which modulate immune responses by enhancing lipolysis and reducing TNF-α production. Mechanistically, they act via cGMP–PKG signaling, attenuating NF-κB–mediated inflammation [95,102,103,104]. The hepatokine FGF21 is upregulated during exercise via PPARα and AMPK pathways. FGF21 suppresses IL-1β and TNF-α secretion from macrophages and enhances Treg function [105]. These effects position FGF21 as a systemic anti-inflammatory exerkine, relevant for metabolic and immune health [95]. (Table 4).

### 2.19. Proposed Framework for Immune-Focused Guidelines

**Figure 2:**
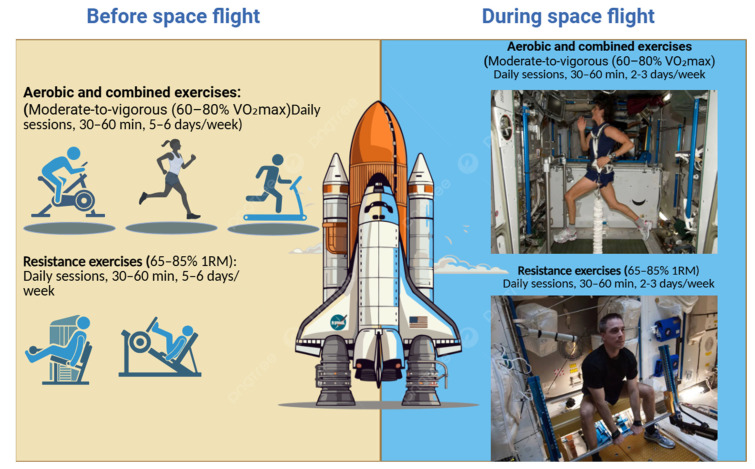
Schematic diagram of an exercise guide focusing on the immune system. In schematic figure, the exercise was added by astronauts from the NASA website. URL (source: https://www.nasa.gov/stem-content/train-like-an-astronaut/, accessed on 26 September 2025). Compared with current musculoskeletal/cardiovascular guidelines, these immune-focused recommendations emphasize moderate intensity, shorter sessions, structured recovery, and immune biomarker monitoring. Figure 2 created by the authors.

Spaceflight-associated immune dysregulation, including impaired lymphocyte activation, latent viral reactivation, and heightened systemic inflammation, underscores the need for countermeasures that extend beyond musculoskeletal and cardiovascular endpoints [1,2,3]. While NASA and ESA currently prioritize resistance and aerobic exercise to mitigate muscle and bone loss, these regimens rarely integrate immune health as an explicit outcome. Given the evidence linking structured exercise to immune modulation, we propose an immune-focused framework for exercise countermeasures in spaceflight [1,2,3]. First, the type of exercise should involve a combination of aerobic, resistance, and high-intensity interval training (HIIT) [14,16]. Aerobic exercise, such as treadmill or cycle ergometer protocols, supports cardiovascular resilience and metabolic balance, while resistance training via the Advanced Resistive Exercise Device (ARED) maintains muscle and bone integrity through PI3K–AKT–mTOR signaling [106]. HIIT may confer unique immunological benefits by stimulating the secretion of exerkines such as IL-15, thereby enhancing natural killer (NK) cell and CD8+ T-cell cytotoxicity, critical for antiviral and antitumor surveillance [66,90]. Second, exercise intensity should be prescribed at a moderate-to-vigorous level (approximately 60–80% VO_2_max for aerobic modalities and 65–85% one-repetition maximum [1RM] for resistance training). This level balances immune enhancement with the avoidance of excessive reactive oxygen species (ROS) generation and post-exercise lymphopenia, which are commonly observed following exhaustive exercise [66,90]. Third, the frequency and duration of exercise should involve daily countermeasure sessions lasting 30–60 min, with 5–6 training days per week [4,38,66,76,88,90,91]. Alternating between resistance-focused and aerobic-focused sessions may maximize systemic adaptations while preventing monotony and overuse injuries. Fourth, personalization of exercise protocols is essential, particularly during long-duration missions. Regular monitoring of immune biomarkers—including cytokine panels (IL-6, IL-10, TNF-α), NK-cell counts, and T-cell subsets—should inform training adjustments to maintain immune resilience. Such individualized feedback loops can minimize risks of overtraining or immunosuppression [4,38,66,76,88,90,91]. Finally, exercise should be integrated with broader lifestyle factors that collectively support immune health. Nutritional strategies—including adequate protein intake to activate mTOR signaling, antioxidants to mitigate oxidative stress, and omega-3 fatty acids to reduce chronic inflammation—should be synchronized with exercise programming. Likewise, sleep optimization and circadian rhythm alignment are critical for hypothalamic–pituitary–adrenal (HPA) axis recovery, while stress management interventions (e.g., mindfulness, heart rate variability monitoring) may further protect against neuroendocrine-induced immune suppression [4,38,66,76,88,90,91]. Taken together, this framework emphasizes a shift from musculoskeletal-centric to immune-inclusive countermeasure design. By integrating exercise type, intensity, duration, personalization, and lifestyle synergy, space agencies can develop a comprehensive protocol aimed at preserving immune surveillance and reducing infection or cancer risks during prolonged missions to the Moon and Mars. (L477: Table 5 and Figure 2).

### 2.20. Influence of Individual Factors on Immune Responses to Spaceflight and Countermeasures

Individual characteristics—including sex, age, and genetic background—exert measurable effects on immune adaptation during spaceflight and may alter the efficacy of exercise-based countermeasures. Sex hormones modulate both innate and adaptive immune function; estrogens enhance NK-cell cytotoxicity, DC–T cell interactions, and antibody production, whereas testosterone is associated with suppression of cellular immunity [107,108,109]. Aging is accompanied by thymic involution, reduced naïve T cell output, impaired B cell repertoire diversity, and a pro-inflammatory cytokine milieu, collectively termed “inflammaging”, which may attenuate the benefits of exercise countermeasures in older astronauts [110]. Furthermore, single nucleotide polymorphisms (SNPs) in immune-relevant genes—such as IL6, TNF, SOD2, and TLR4—have been linked to interindividual variability in oxidative stress resistance, cytokine responses, and susceptibility to latent viral reactivation, thereby influencing countermeasure outcomes [111]. The integration of sex-, age-, and genotype-specific data into study designs will enable the development of precision exercise prescriptions that optimize immune resilience during exploration-class missions.

### 2.21. Future Directions and Research Gaps

Despite accumulating evidence linking spaceflight to immune dysregulation, significant knowledge gaps remain regarding the role of exercise as an immunoprotective countermeasure. To date, most investigations aboard the International Space Station (ISS) have focused on musculoskeletal and cardiovascular outcomes, while randomized controlled trials incorporating immune endpoints are virtually absent. Future studies should be designed to evaluate immune-specific markers—such as NK-cell cytotoxicity, T-cell proliferation, cytokine panels, and latent viral reactivation—within structured exercise protocols in astronauts. Moreover, the development of standardized immune monitoring protocols on the ISS is urgently needed. Portable flow cytometry, multiplex cytokine assays, and real-time viral load detection are now technically feasible and could provide actionable insights for tailoring individualized countermeasures in-flight. Recent advances in wearable biosensors and lab-on-a-chip systems offer complementary real-time monitoring capabilities that could synergize with portable flow cytometry for in-flight immune assessment. For example, the APHRODITE project demonstrated a compact, reusable chemiluminescence-based lab-on-chip device capable of detecting salivary biomarkers such as cortisol and DHEA-S aboard the ISS, enabling stress and immune status monitoring without bulky instrumentation [112]. Likewise, recent reviews highlight the potential of wearable sensor platforms integrating physiological and biochemical sensing for continuous astronaut health surveillance in extreme environments [113]. Integration of such biosensing technologies into spaceflight health protocols could facilitate earlier detection of immune dysregulation, improve biomarker trend analysis, and reduce crew time requirements during long-duration missions. Another promising avenue is the integration of exercise with pharmacological agents. For example, antioxidant supplementation may mitigate exercise-induced ROS, while immunomodulatory agents (e.g., IL-7 or IL-15 analogs) could synergize with exercise to preserve immune resilience. Similarly, combining resistance training with bisphosphonates or selective androgen receptor modulators (SARMs) could yield dual musculoskeletal and immune benefits [114]. Finally, the emerging role of exerkines and metabolites such as lactate in immune regulation highlights a novel research direction. Molecules such as IL-15, irisin, and lactate not only serve as biomarkers of exercise intensity but may also act as mechanistic mediators of immune adaptation in microgravity. Including the modulation of metabolic intermediates such as lactate, which is increasingly recognized not only as a by-product of anaerobic metabolism but also as an important signaling molecule in exercise immunology [114]. During acute, moderate-intensity activity, transient increases in lactate can serve as a metabolic cue that supports immune cell energy demands and enhances macrophage phagocytic capacity, lymphocyte proliferation, and cytokine release. These effects may contribute to improved immune surveillance and faster resolution of inflammation. Conversely, chronically elevated lactate levels—such as those observed during exhaustive exercise or in certain pathophysiological conditions—have been shown to promote local immunosuppression, in part through the inhibition of monocyte antigen-presenting functions, suppression of NK cell cytotoxicity, and alteration of T-cell metabolism [114]. This dual role underscores the importance of optimizing exercise intensity and recovery to harness the beneficial immunomodulatory potential of lactate while avoiding its deleterious effects. Elucidating their signaling pathways in space physiology may uncover new therapeutic targets and refine biomarker-based monitoring of astronaut health. Future research should systematically evaluate antioxidant-based countermeasures—both nutritional and exercise-induced—against microgravity-driven oxidative stress. Ground-based analogs and ISS longitudinal sampling indicate that ROS scavenging pathways (e.g., superoxide dismutase, catalase, GPX1) are variably modulated in flight, and their optimization could mitigate immune suppression and chronic inflammation risks in long-duration space missions [11,12,13].

## 3. Conclusions

Exercise remains the cornerstone countermeasure in spaceflight, effectively mitigating muscle atrophy, bone loss, and cardiovascular deconditioning. However, current exercise guidelines are incomplete, as they rarely account for the profound alterations in immunity that threaten astronaut health during long-duration missions. By shifting the paradigm toward an immune-inclusive framework, exercise can be strategically harnessed to enhance antiviral surveillance, improve adaptive immune function, and reduce systemic inflammation. The proposed framework—integrating exercise modality (aerobic, resistance, HIIT), intensity prescription, immune monitoring, and lifestyle factors such as nutrition and sleep—offers a comprehensive approach to astronaut healthcare. Incorporating immune-focused countermeasures will not only improve astronaut safety and resilience during missions to the Moon and Mars but also advance terrestrial medicine by revealing novel insights into exercise immunology. Ultimately, recognizing immunity as a core endpoint in space physiology will be essential for the success of future exploration-class missions. Pharmaceutical agents may be used in combination with exercise to mitigate oxidative stress. Nutritional strategies—such as antioxidant-rich diets (e.g., polyphenols from berries, catechins from green tea), omega-3 fatty acids, and adequate micronutrient intake (e.g., vitamins C and E, selenium, zinc)—also exert protective effects against excessive ROS generation and inflammation, and may enhance the efficacy of exercise-based countermeasures during spaceflight.

## Figures and Tables

**Table 1:** Summary of immune dysregulation in spaceflight.

Immune Component	Effects of Microgravity	Clinical/Functional Outcomes	Potential Exercise Link
Viral Reactivation [1,20,21]	Suppression of cellular immunity; ↑ cortisol, epinephrine, norepinephrine	Reactivation of EBV, CMV, VZV; higher prevalence in long missions	Moderate exercise may improve stress hormone regulation & viral surveillance
Dendritic Cells (DCs) [25,26,27]	Impaired maturation; ↓ IL-2 secretion	Weakened T-cell activation; ↑ autoimmunity risk	Exercise enhances DC–T cell interaction terrestrially
Neutrophils [22,28,29,30,31]	↑ circulating counts; impaired phagocytosis & oxidative burst	Reduced host defense; ↑ NLR (marker for cancer/inflammation)	Aerobic/resistance training shown to normalize NLR
NK Cells [9,32,33]	↓ survival & cytotoxic activity; ↑ apoptosis	Impaired tumor & viral surveillance	Moderate aerobic exercise enhances NK activity on Earth
B Cells [34,35,36,37]	↓ abundance (animal models); ↑ apoptosis (oxidative stress)	Reduced antibody production; impaired humoral immunity	Exercise enhances antibody response to vaccination
T Cells [3,36,39,41,42].	↓ proliferation & activation (CD25, CD69, IL-2, IFN-γ); altered JAK–STAT, NF-κB	Suppressed adaptive immunity; ↑ Treg activity	Exercise boosts T-cell function & cytokine balance
Metabolism/Structure [5,43,44,45,46,47,48,49]	Altered lipid/nucleotide metabolism; impaired cytoskeleton (F-actin, microtubules)	↓ motility, phagocytosis, antigen presentation	Exercise regulates metabolism & ROS handling
Radiation Exposure [52,53,54].	DNA damage from HZE particles; ↑ carcinogenesis risk	Elevated lifetime cancer risk, esp. for long-duration missions	Exercise may mitigate oxidative stress & DNA repair efficiency

**Table 2:** Summary of countermeasures (exercise) in spaceflight.

Device/Protocol	Primary Purpose	Physiological Outcomes	Evidence Strength	Identified Gaps
ARED (Advanced Resistive Exercise Device) [14,19,63,66,67,68,69,70,71]	Replicates resistance training with concentric & eccentric loads	↑ Muscle mass, ↑ Strength, Prevents bone loss (via PI3K–AKT–mTOR signaling)	Strong (flight & ground analogs, 16-week studies)	No immune endpoints measured; unclear effect on inflammation/cytokines
Treadmill (with Subject Load Devices) [72,73]	Simulated gravity via tethered running/walking	Maintains cardiovascular function, reduces musculoskeletal deconditioning	Moderate to strong (decades of implementation)	Studies focus on VO_2_max & muscle; little on immune modulation
Cycle Ergometer [75]	Aerobic conditioning with partial resistive load	Improves cardiovascular fitness, supports muscle endurance	Moderate (supported by ISS studies)	No systematic investigation of immune cell function or cytokine responses
Combined Aerobic + Resistance Training [76,77,78]	Integrates multiple modalities	Enhances hypertrophy & cardiorespiratory performance	Growing (meta-analyses support Earth-based benefits)	No studies in astronauts assessing combined effects on immunity

**Table 3:** Immunological changes (acute and chronic exercise).

Feature	Acute Exercise	Chronic Exercise
Leukocytes	Rapid mobilization of NK, CD8+ T, neutrophils via β2-adrenergic signaling [82,85,86,87,88]	Long-term enhancement of NK cytotoxicity and T-cell homeostasis [89]
Cytokines	↑ IL-6, TNF-α, IL-1β (transient); IL-10 counter-regulation [82]	↓ Baseline TNF-α, IL-6, CRP; ↑ IL-10 [88]
Signaling Pathways	NF-κB, MAPK, STAT3 transiently activated [82]	Downregulation of NF-κB, sustained AMPK–mTOR balance [88]
ROS/Redox	Acute ↑ ROS → transient oxidative stress [87].	↑ Antioxidant enzymes (SOD, GPx) [89]
Hormones	Acute ↑ cortisol, catecholamines [82]	HPA axis adaptation → reduced basal cortisol [90]
Immunosenescence	No effect (short-term) [88].	Delayed immunosenescence, preserved TCR diversity [89]

**Table 4:** Immunological changes (Exerkines).

Exerkine	Source	Signaling Pathways	Immune Effects
IL-6 [82,93]	Muscle	NF-κB, p38 MAPK, JAK–STAT3	↑ IL-10, IL-1ra; ↓ TNF-α; NK trafficking
IL-7 [82,93,96]	Muscle	JAK–STAT5	Thymopoiesis, naïve T-cell survival
IL-15 [90]	Muscle	JAK–STAT3/5	NK proliferation, CD8+ T-cell memory
Irisin [95,97]	Muscle (FNDC5 cleavage)	AMPK–PGC1α, NF-κB inhibition	↓ Macrophage TNF-α; ↑ Tregs
Myostatin [95,97]	Muscle	TGF-β–SMAD2/3	Fibrosis, immune suppression (↓ with exercise)
Leptin [95,98]	Adipose	JAK2–STAT3, PI3K–AKT	↑ Th1 cytokines, pro-inflammatory
Adiponectin [99]	Adipose	AMPK–PPARα	↓ NF-κB, ↑ anti-inflammatory
FGF21 [105]	Liver	AMPK, PPARα	↓ IL-1β/TNF-α; ↑ Tregs
BNP/ANP [105]	Heart	cGMP–PKG	↓ NF-κB, ↓ TNF-α
BDNF [95,98]	Brain	TrkB–MAPK, PI3K	↑ NK activity, anti-inflammatory macrophages

**Table 5:** Physical activity guidelines for astronauts (with a focus on immunological changes)—(Differences from current musculoskeletal/cardiovascular guidelines are highlighted in accompanying text.).

Parameter	Recommendation	Immune Rationale
Type of Exercise [4,38,66,76,88,90,91]	Combined aerobic (treadmill/cycle), resistance (ARED), HIIT and Resistance training	Synergistic effects on cardiovascular, musculoskeletal, and immune systems (IL-15, NK activation)
Intensity [4,38,66,76,88,90,91]	Moderate-to-vigorous (60–80% VO_2_max; 65–85% 1RM)	Optimizes immune protection while avoiding ROS overload and post-exercise lymphopenia
Frequency/Duration [4,38,66,76,88,90,91]	Daily sessions, 30–60 min, 5–6 days/week	Maintains immune surveillance, reduces viral reactivation, prevents immune decline
Personalization [4,38,66,76,88,90,91]	Immune monitoring (cytokines: IL-6, IL-10, TNF-α; NK/T-cell counts)	Enables adjustment of exercise load according to immune status and resilience
Lifestyle Integration [4,38,66,76,88,90,91]	Nutrition (antioxidants, omega-3s, protein), sleep optimization, stress control	Supports HPA axis recovery, reduces chronic inflammation, enhances immune regulation

## Data Availability

Data will be made available upon reasonable request.

## Abbreviations

ARED	Advanced Resistive Exercise Device
BBB	Blood–Brain Barrier
BDNF	Brain-Derived Neurotrophic Factor
CMV	Cytomegalovirus
CRP	C-Reactive Protein
DC	Dendritic Cell
EBV	Epstein–Barr Virus
FGF21	Fibroblast Growth Factor 21
HPA axis	Hypothalamic–Pituitary–Adrenal axis
HIIT	High-Intensity Interval Training
IL	Interleukin
ISS	International Space Station
MAPK	Mitogen-Activated Protein Kinase
mTOR	Mechanistic Target of Rapamycin
NASA	National Aeronautics and Space Administration
NK cell	Natural Killer cell
NLR	Neutrophil-to-Lymphocyte Ratio
PI3K–AKT	Phosphoinositide 3-Kinase–Protein Kinase B Pathway
PPARα	Peroxisome Proliferator-Activated Receptor Alpha
ROS	Reactive Oxygen Species
RTE	Reentry
SASP	Senescence-Associated Secretory Phenotype
SLDs	Subject Load Devices
STAT	Signal Transducer and Activator of Transcription
TCR	T-Cell Receptor
TLR4	Toll-Like Receptor 4
TNF-α	Tumor Necrosis Factor Alpha
URTI	Upper Respiratory Tract Infection
VZV	Varicella–Zoster Virus
VO_2_max	Maximal Oxygen Uptake
